# Jornal de Pediatria at a turning point: four years of growth and the road ahead in the age of AI

**DOI:** 10.1016/j.jped.2025.101481

**Published:** 2025-12-04

**Authors:** Renato Soibelmann Procianoy

**Affiliations:** a Full Professor of Pediatrics, Universidade Federal do Rio Grande do Sul (UFRGS), Porto Alegre, RS, Brazil; b Full Member of the Brazilian Academy of Pediatrics, Rio de Janeiro, RJ, Brazil; c Editor-in-Chief, Jornal de Pediatria, Porto Alegre, RS, Brazil

Over the last years, Jornal de Pediatria has moved from being primarily a national reference for Brazilian pediatrics to occupying a more visible and respected space within the international pediatric literature. This transformation has not been accidental. It reflects deliberate editorial choices, robust work from Brazilian researchers, and a strategic alignment with global trends in scientific publishing — open access, methodological rigor, and digital dissemination. At the same time, the Brazilian Society of Pediatrics (Sociedade Brasileira de Pediatria, SBP) has created new bridges between the journal and its members, most notably through the Reportes Semanais (“Weekly Reports”), a weekly newsletter that has become one of the most dynamic tools for engaging the pediatric community with the journal’s content.

As we look forward, another powerful force is reshaping the landscape: artificial intelligence (AI). AI is already transforming pediatric care and research, and it will inevitably transform how we write, review, edit, and read scientific publications. The question is not whether journals like Jornal de Pediatria will adapt, but how—and on whose terms.

## The evolution of Jornal de Pediatria in the last four years within the international pediatric literature

From a metrics standpoint, the past four years have been among the most significant in the journal’s history. According to its official metrics, Jornal de Pediatria saw its impact factor rise from 2.2 in 2020 to 3.0 in 2021 and reach 3.3 in 2022, before adjusting to 2.8 in 2023 [1]. Data from SBP highlight that the 2022 Impact Factor placed Jornal de Pediatria roughly in the 75th percentile among pediatric journals indexed in major databases, meaning that about three quarters of listed pediatric titles had lower reach [2]. More recent recalculations indicate that the Jornal de Pediatria is now ranked in the first quartile of international pediatric journals (78.8th percentile) [3].

These numbers are not merely symbolic trophies. They reflect an increased volume of citations to Brazilian pediatric research and a growing presence of Jornal de Pediatria articles in international discussions on child health, from infectious diseases and vaccination to chronic illnesses, mental health, and social determinants of health. The journal is fully online, bimonthly, and published by SBP, with indexing and hosting through SciELO and distribution via platforms such as ScienceDirect, which places its content side-by-side with leading international pediatric titles [4].

Thematically, the last four years have coincided with a period of intense pressure on pediatric services worldwide, driven initially by the COVID-19 pandemic and its pediatric manifestations. Jornal de Pediatria published key contributions on COVID-19 in children, including work on the multisystem inflammatory syndrome and the distinct clinical profile of pediatric cases, which were widely cited in both Brazilian and international literature. As the acute phase of the pandemic eased, the journal’s portfolio diversified further, covering long-term developmental and mental health consequences, inequities in access to care, and contemporary challenges such as obesity, digital media exposure, and vaccine hesitancy.

Structurally, the journal has also modernized. It is a fully open-access publication, with articles immediately available under open licenses [5]. In early 2025, Jornal de Pediatria announced changes in the article publishing charges (APCs) for accepted manuscripts, aligning with global business models for open-access journals while maintaining free submission [6]. The decision brings Jornal de Pediatria into the same conversation as many high-impact international pediatric journals that rely on APCs, but it also poses the challenge of ensuring that this transition does not create barriers for authors from less-resourced settings.

In short, when we compare Jornal de Pediatria today with its profile four years ago, we see a journal that is more cited, more visible, more international in reach, and more fully integrated into the global ecosystem of pediatric publishing—while still maintaining its identity as the voice of Brazilian pediatrics.

## The weekly reports and their impact on dissemination among SBP members

If metrics are one dimension of growth, another is the quality of the relationship between the journal and its primary community: the pediatricians of Brazil. Here, the Reportes Semanais of Jornal de Pediatria has been a quiet revolution.

Reportes Semanais is a weekly newsletter highlighting selected content from the journal distributed by email to SBP members and also made available on the SBP website [7]. Announcements from SBP describe it as a “new project” of the journal, with successive issues being released and explicitly promoted to members as essential reading [8].

While we may not yet have formal readership surveys or click-through analytics publicly available, it is reasonable to say that the Reportes Semanais has likely increased the journal’s visibility within SBP.

## The coming wave: artificial intelligence and the future of scientific publishing

Artificial intelligence is no longer a distant promise in pediatrics; it is already influencing clinical care, research, and health policy. AI tools are being explored to diagnose sepsis, analyze pediatric imaging, predict outcomes in intensive care, and support decisions in complex chronic conditions. Recent editorials and position papers in pediatrics emphasize both the extraordinary potential of AI to improve efficiency and quality of care and the profound ethical and governance challenges it raises [9].

In the realm of scientific publishing, AI is already changing how manuscripts are produced and evaluated. Large language models can help authors draft, translate, and polish manuscripts; machine-learning tools can screen submissions for plagiarism, statistical anomalies, and image manipulation; and automated systems can assist editors in identifying suitable reviewers or flagging potential conflicts of interest. At first glance, these tools promise faster, more efficient workflows and lower barriers for researchers whose first language is not English.

For a journal like Jornal de Pediatria, AI offers several concrete opportunities:

A. *Improved language support for authors*
  Many Jornal de Pediatria authors write in English as an additional language. With clear policies and guidance, AI-assisted language tools could help make manuscripts clearer and more accessible, without compromising scientific integrity or masking conceptual weaknesses.
B. *Smarter manuscript triage*
  AI could support editors in the initial screening of submissions, helping to identify papers that clearly fall outside the journal’s scope, have glaring methodological flaws, have been published elsewhere, have a high plagiarism index, and have already been submitted to the Jornal de Pediatria or another journal, thus, conserving human reviewer time for more nuanced evaluations.
C. *Enhanced discoverability and summarization*
  AI tools could generate structured summaries, visual abstracts, and multilingual lay summaries of published articles, which might enrich Reportes Semanais, SBP newsletters, and social media, and help non-specialist clinicians access key messages more quickly.
D. *Better linkage between clinical practice and research*
  By mining Jornal de Pediatria’s archive and cross-linking it to guidelines, SBP statements, and other national documents, AI systems could help clinicians identify the most relevant Jornal de Pediatria articles for specific clinical questions, reinforcing the journal’s role as a practical resource.

However, these opportunities come with serious risks. AI systems can “hallucinate” references, invent data, and reproduce biases present in their training sets.

For scientific journals, this means that AI cannot be treated as a neutral helper. It must be governed.

## Future perspectives for Jornal de Pediatria in the age of AI

Looking ahead, several strategic directions emerge for Jornal de Pediatria:

A. *Strengthening its role in global pediatrics.*
  The last four years have shown that Brazilian pediatric research is not just locally relevant; it is globally impactful. With its open-access model and established indexing, Jornal de Pediatria is well positioned to become a leading platform for pediatric research [2].
B. *Developing a clear AI strategy for publishing.*
  Rather than reacting piecemeal to AI-related challenges, Jornal de Pediatria could craft a coherent strategy that covers:
  * internal workflows (screening, peer-review support, production);
  * guidance for authors and reviewers;
  * ethical standards for AI-related research;
  * and communication to readers about how the journal ensures integrity in an AI-rich environment.

This strategy should align with emerging international guidelines on AI in health research and build on the growing pediatric-specific literature on responsible AI.

## Conclusion: A journal ready for its next chapter

The last four years have been a period of acceleration for Jornal de Pediatria. The journal’s rising impact factor, solid position among international pediatric titles, and innovative initiatives such as the Reportes Semanais attest to a publication that is not content to stand still. It is moving—toward greater visibility, broader influence, and deeper engagement with the pediatric community it serves [2].

At the same time, the horizon is filled with new challenges. AI will transform how we generate, evaluate, and disseminate knowledge. For some journals, this transformation may simply be something that “happens to them.” For Jornal de Pediatria, it can be a transformation that is actively shaped: guided by the values of pediatric care, by SBP’s commitment to children and families, and by a clear understanding that technology is a tool — not a substitute — for scientific rigor and ethical responsibility.

If the last four years have shown anything, it is that Brazilian pediatrics and Jornal de Pediatria are capable of competing, contributing, and leading on the global stage. The task now is to extend this leadership into the AI era: to embrace new tools without losing our critical sense; to speed up workflows without sacrificing depth; and to use every innovation — editorial, educational, technological — to bring high-quality pediatric evidence closer to those who need it most: the clinicians at the bedside and, ultimately, the children and adolescents of Brazil and the world.

## Funding

This research received no external funding.

## Data availability statement

Not applicable.

## Declaration of competing interest

The author declares no conflicts of interest.
